# Design Key Points of High-Performance Diffuse Reflectance Optical Sensors for Non-Invasive Blood Glucose Measurement

**DOI:** 10.3390/s25040998

**Published:** 2025-02-07

**Authors:** Wenbo Liu, Tongshuai Han, Wenliang Chen, Jiayu Chen, Qing Ge, Di Sun, Jin Liu, Kexin Xu

**Affiliations:** 1 State Key Laboratory of Precision Measurement Technology and Instruments, Tianjin University, Tianjin 300072, China; wenbo_liu@tju.edu.cn (W.L.); hts2014@tju.edu.cn (T.H.); chenwenliang@tju.edu.cn (W.C.); jiayu_chen@tju.edu.cn (J.C.); geqing@tju.edu.cn (Q.G.); sundi@tju.edu.cn (D.S.); 2 Sunrise Technology Co., Ltd., Tianjin 300192, China

**Keywords:** optical sensor, design key points, non-invasive blood glucose measurement, Monte Carlo simulation

## Abstract

Optical sensors serve as pivotal components in the realm of non-invasive blood glucose measurement (NBGM) devices, where their efficacy directly influences the detection of weak glucose signals. This research introduces three fundamental design key points tailored for diffuse reflectance optical sensors employed for NBGM: depth resolution, detection signal-to-noise ratio, and human–sensor interface coupling. Guided by these design key points, we presented feasible design proposals for near-infrared diffuse reflectance sensors operating in the range of 1000–1700 nm. As an example, a sensor composed of five-ring detectors with a ring-shaped mask were made and tested on human skin. The innovative sensor developed herein holds promising potential for NBGM.

## 1. Introduction

When using diffuse reflectance spectroscopy for non-invasive blood glucose measurement (NBGM) in humans, the measurement signals are often weak and unstable. To perform NGBMs, high-performance sensors are necessary. In addition, stable measurement conditions need to be established, including environmental factors (such as temperature and humidity, etc.), human–sensor interface factors (such as contact pressure and measurement posture, etc.), and physiological background states of the human body (such as blood flow, water content, etc.). Numerous studies have proposed methods to ensure stable measurement conditions and have achieved practicable results [1,2,3,4,5,6,7,8]. In this paper, three important design points that must be considered are proposed and analyzed when designing sensors for NGBM.

To design optical sensors capable of meeting the resolution requirements for these weak blood glucose concentration (BGC) signals, specific performance criteria must be satisfied. Firstly, human skin has a naturally layered structure with an uneven distribution of components across different layers [9]. Thus, the sensor must possess the necessary depth resolution to accurately capture changes in information at varying depths. Secondly, given the inherently weak signal of BGC obtained through diffuse reflectance spectroscopy [10], the sensor must achieve a sufficient detection signal-to-noise ratio (SNR) to effectively differentiate the target signal from background noise. Finally, due to the softness and deformability of human skin, a stable and reliable coupling between the skin and sensor is essential to ensure accurate data acquisition during wear. Based on these considerations, we propose three design key points for diffuse reflectance optical sensors: depth resolution, detection SNR, and human–sensor interface coupling [11,12,13]. These design key points must meet their respective requirements. The following sections elaborate on these key points:

(1) Depth resolution

Depth resolution refers to the sensor’s ability to precisely identify changes within a specific depth of tissue. BGC detection often targets the dermis, which has a high water content and relative uniformity [14,15]. Multi-wavelength measurements are commonly employed, and it is critical to ensure that the measurement depth ranges of the selected wavelengths fall within the dermis as much as possible. However, differences in penetration depths among wavelengths, along with variability in skin optical parameters and thickness across subjects and body regions, necessitate the design of multiple detection depths to meet diverse requirements. Additionally, changes in cutaneous blood perfusion can interfere with NBGMs [16]. Therefore, the sensor design must address the detection needs of thin tissue layers, such as the upper and deeper blood net dermis [17]. These requirements demand a sensor with a depth resolution of targeting specific tissue layers across multiple wavelengths. In practical applications, optical fibers placed at different source-detector separations (SDSs) are typically used for light reception to measure the change in different tissue depths [18,19].

(2) Detection SNR

Detection SNR refers to the sensor’s ability to reliably detect and distinguish weak target signals from noise. In this study, the SNR specifically refers to the repeatability for the measurement directly on tissue. The SNR can be calculated by the ratio of the mean to the standard deviation of the light intensity. To distinguish the change of 1 mM BGC, its signal amplitude must be greater than the noise level. Generally, using optical fiber bundles arranged in a ring-shaped structure can improve the sensor’s SNR [20]. However, optical fibers have a certain numerical aperture, and losses may occur during the transmission process. Some groups have also employed a single photodiode placed close to the skin to directly receive light, which helps enhance the SNR [21,22]. Ge et al. [10] proposed using a ring-shaped photodiode placed close to the skin to directly receive light for NBGMs. At 1550 nm, the BGC sensitivity is 0.001 a.u./mM for the SDS of 2.0 mm. And, at 1550 nm, the BGC sensitivity is −0.002 a.u./mM for differential SDSs of 2.0 and 2.6 mm. Therefore, to resolve a 1 mM change in the BGC, a SNR of at least 1000:1 is needed at a SDS of 2.0 mm. If differential measurements are using SDSs of 2.0 mm and 2.6 mm, the differential equivalent SNR must be at least 500:1. To achieve a BGC resolution better than 1 mM, a sensor with a higher SNR is required.

(3) Human–sensor interface coupling

Human–sensor interface coupling refers to the sensor’s ability to stably and effectively receive light from tissues that may be in motion for long-term monitoring. A wearable sensor must maintain stable and accurate detection, despite tissue deformation and physiological changes. Even if a sensor meets the requirements for the depth resolution and detection SNR, challenges related to human–sensor interface stability can arise in practical applications. This study focuses on the interface coupling between the skin and sensor. Our research group developed a sensor using InGaAs photosensitive materials [8], achieving a short-term (1 min) SNR between 7000:1 and 10,000:1 on a diffuse reflectance standard at 1550 nm. When worn on the human body, the short-term (1 min) SNR ranged between 1000:1 and 5000:1, a reduction of 2–10 times compared to the sensor’s intrinsic performance. Over 30 min of wear, the SNR further decreased to between 300:1 and 800:1 due to minor movements affecting the coupling interface. To mitigate this issue, various strategies have been employed. Tronstad et al. [23] monitored skin temperature and used straps to control skin contact pressure for stable spectra collection. Aloraynan et al. [24] used mid-infrared quantum cascade lasers (QCLs) and maintained skin dryness by flowing nitrogen gas to prevent humidity and sweat accumulation from affecting the measurements. Our research group developed a miniaturized, ring-shaped sensor that achieved a good enough detection SNR on the human body while addressing certain skin–sensor interface instability issues through the differential measurement strategy. Using our research group’s sensor, Han et al. [8] achieved preferable results by stably and directly detecting BGC signals at a single wavelength. However, challenges such as spatial crosstalk and sweat-induced signal changes persist, necessitating further improvements.

In summary, this paper systematically examines three necessary key points essential for the design of diffuse reflectance optical sensors for NBGM: depth resolution, detection SNR, and human–sensor interface coupling. We present design proposals for each of these design key points. Using the Monte Carlo eXtreme (MCX) tool, which is capable of simulating complex human–sensor interfaces, a simulation-based analysis was conducted to investigate how sensors can achieve these key points within a seven-layer skin structure. Based on the simulation results, an optical sensor was proposed, and its performance was preliminarily tested on human forearm skin. This study is expected to provide references for the design of NBGM sensors.

## 2. Design Key Points of Diffuse Reflectance Optical Sensors

To accurately extract BGC signals, the diffuse reflectance optical sensor should follow the three design key points and requirements in Table 1. For each key point, this study provides some design proposals. The following sections evaluate these proposals using both simulated and experimental data.

We define three performance parameters for sensors: detection SNR, BGC sensitivity, and limit of detection for the BGC. The first two parameters determine the sensor’s limit of detection. The following will be the calculation and derivation of these parameters.

(1) Detection SNR

The detection SNR primarily indicates the sensor’s noise level, and it is defined as the ratio of the mean light intensity, $\bar{I}$, to the standard deviation, σI, within a given time range, as shown in Equation (1) [25].

(1) $$SNR=\left|\frac{\bar{I}}{\sigma I}\right|$$

(2) $$\sigma I=f(\sigma I_{detector},\;\sigma I_{human})$$

In the near-infrared band, σI is primarily composed of the detector’s thermal noise ($\sigma I_{detector}$) and the human noise ($\sigma I_{human}$), as shown in Equation (2). To enhance a sensor’s SNR, the light intensity, I, can be increased while simultaneously reducing the noise, σI.

In near-infrared band, $\sigma I_{detector}$ mainly is the detector’s thermal noise. Therefore, controlling the temperature of the detector can stabilize the level of thermal noise. Meanwhile, the level of light intensity can be increased by increasing the detection area. Generally, as the detector area increases, the SNR becomes higher. $\sigma I_{human}$ is the noise generated during human measurement, which is mainly related to factors such as the jitter of the measurement site and the non-uniform skin surface reflection. Typically, $\sigma I_{human}\gg \sigma I_{detector}$, meaning human noise is dominant in the overall noise (σI). The noise caused by measurement site jitter is typically random. Increasing the detection area also can suppress such random noise by averaging it. The skin surface reflection light is influenced by the skin’s surface morphology and its random jitter, which may exhibit anisotropic interference. To reduce $\sigma I_{human}$, we made various attempts, for example, designing the detector as a ring shape—ring-shaped light reception is beneficial for averaging the anisotropic change over a 360 degree range, further suppressing noise [8].

The absorbance is defined as:

(3) $$A\left(\lambda ,\rho \right)=-ln\left(\frac{I(\lambda ,\rho )}{I_{0}(\lambda )}\right)$$

where λ represents the measurement wavelength, ρ represents the source-detector separation (SDS), I represents the intensity of the diffuse reflectance light, and $I_{0}$ represents the intensity of the incident light. The noise level of absorbance, which is the standard deviation over a given time period, is denoted as σA. By differentiating Equation (3), we can conclude: σA=σI/I. Therefore, Equation (1) can be rewritten as Equation (4). It means that a lower σA results in a higher SNR.

(4) $$SNR=\left|\frac{1}{\sigma A}\right|$$

(2) BGC sensitivity

BGC sensitivity refers to the change in the light signal caused by a unit, like a 1 mM BGC change. In this paper, it specifically refers to the change in absorbance, denoted as S. BGC sensitivity can be described by Equation (5).

(5) $$S=\frac{dA\left(\lambda ,\rho \right)}{dC_{g}}$$

where $C_{g}$ denotes the BGC.

(3) The limit of detection for the BGC

The detection SNR and BGC sensitivity are the two factors that determine the sensor’s ability to distinguish the limit of BGC ($C_{g,limit}$), which can be expressed as:

(6) $$C_{g,limit}=\frac{1}{\left|S\cdot SNR\right|}=\left|\frac{\sigma A}{\frac{dA\left(\lambda ,\rho \right)}{dC_{g}}}\right|$$

The performance of a sensor will ultimately be evaluated by $C_{g,limit}$. Once the BGC sensitivity is determined experimentally or through a simulation, the SNR is the key factor for the desired $C_{g,limit}$, as follows:

(7) $$SNR\geq \frac{1}{\left|S\cdot C_{g,limit}\right|}$$

## 3. Design Proposals and Evaluations

### 3.1. Design Proposal for Depth Resolution

#### 3.1.1. Multi-SDSs for Various Detection Depths

**Figure 1:**
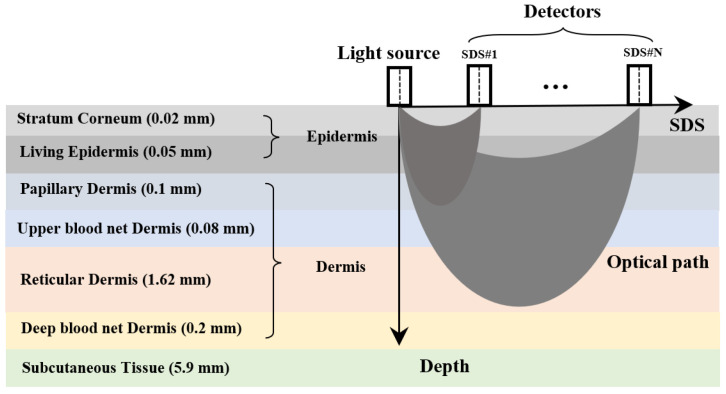
Schematic diagram of the diffuse reflectance measurement method.

A schematic diagram of the diffuse reflectance measurement method is shown in Figure 1 [26]. The multi-SDS design enables the detection of information from specific depth ranges within the skin. The skin is divided into seven layers: the stratum corneum and living epidermis are collectively referred to as the epidermis, while the papillary dermis, upper blood net dermis, reticular dermis, and deep blood net dermis are collectively referred to as the dermis [27]. The thicknesses of these seven skin layers are based on the research from Zherebtsov et al. [28], representing average values across multiple individuals and body regions.

In simulation calculations, cumulative light energy is commonly used to represent light intensity. Thus, Equation (3) can be rewritten as:

(8) $$A\left(\lambda ,\rho \right)=-ln\left(\frac{I(\lambda ,\rho )}{I_{0}(\lambda )}\right)=-ln\left(\frac{\sum_{i=1}^{N}w_{i}(\lambda ,\rho )}{w_{0}(\lambda )}\right)$$

where $w_{0}$ represents the incident light energy, $w_{i}$ is the light energy of the i-th photon received by the detector, and *N* is the total number of photons received by the detector. The mean optical path is defined as follows:

(9) $$\bar{L}=\frac{\sum_{i=1}^{N}w_{i}\cdot L_{i}}{\sum_{i=1}^{N}w_{i}}$$

where $L_{i}$ is the optical path of the i-th photon. The proportion of the optical path of a certain layer to the total optical path is described by the following formula:

(10) $$\beta_{layer}=\frac{\bar{L_{layer}}}{\bar{L}}$$

where $\bar{L_{layer}}$ is the mean optical path in a certain layer. We can use $\beta_{Epidermis}$, $\beta_{Dermis}$, $\beta_{Subcutaneous}$ to represent the contribution of the optical path to the total optical path of the epidermis, dermis, and subcutaneous tissue, respectively. To ensure that the collected photons predominantly pass through the target layer, it is crucial to maximize the β contribution from that layer.

#### 3.1.2. Multi-SDSs for Detection at the Dermis

The Monte Carlo (MC) simulation was employed to model the light transmission process within the skin. This approach allows the determination of the exit light intensity at different SDSs, the mean optical path of the exiting light, and the optical path distribution across each skin layer [29].

The MC simulation is a robust technique for tracing photons in scattering media. In this study, the Monte Carlo eXtreme (MCX) tool was used for the simulation, which offers flexibility in designing complex human–sensor interfaces [30]. The total number of incident photons was 10^8^, with each photon assigned a weight of 1 a.u. The skin dimensions were 8 mm by 8 mm. The incident light was directed from the center of the skin. The method proposed by Yao et al. [31] for calculating the diffuse optical tomography Jacobians using photon “replay” was applied. The strength of the optical field at each spatial position is represented by the product of the optical path and the energy passing through that position.

**Figure 2:**
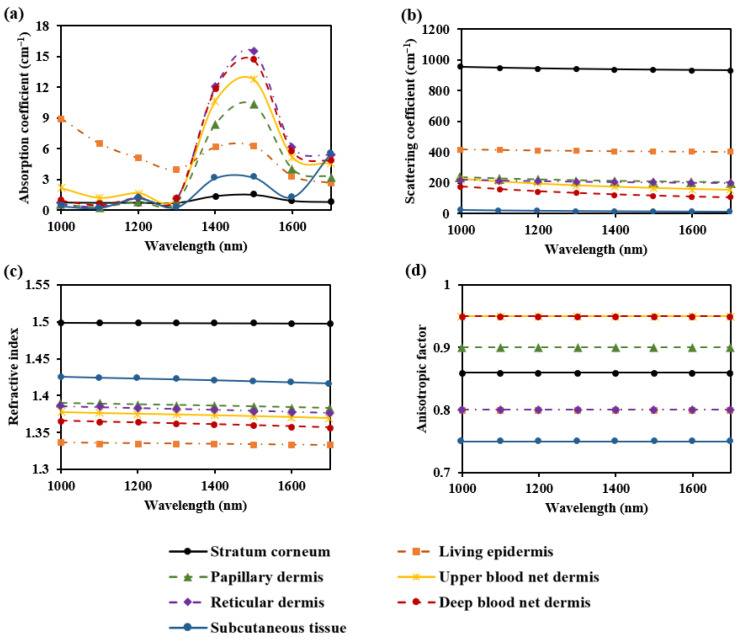
Optical parameters of the seven skin layers: (**a**) absorption coefficient; (**b**) scattering coefficient; (**c**) refractive index; and (**d**) anisotropic factor.

The absorption coefficients for the seven skin layers were determined by multiplying the absorption coefficients of various components (such as blood, interstitial fluid, melanin, fat, and collagen) by their respective volume fractions [17,32,33,34]. The scattering coefficients for the skin layers were obtained using values reported by Meglinski et al. [35] at 633 nm, in combination with the wavelength-dependent distribution curve from Troy et al. [36]. The refractive index for the skin layers was derived from the values reported by Meglinski et al. [35] at 633 nm, in combination with the Cauchy fitting formula from Troy et al. [36]. The anisotropy factor for the skin layers was obtained using values reported by Meglinski et al. [35]. The optical parameters of the seven skin layers across the 1000–1700 nm wavelength range are shown in Figure 2.

**Figure 3:**
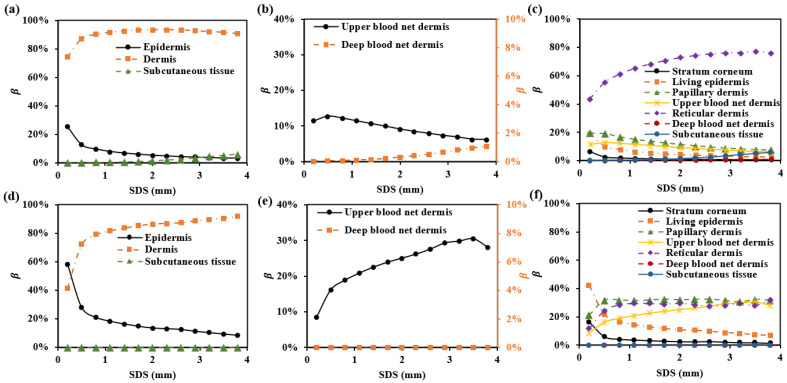
β in each skin layer for multi-SDSs: (**a**–**c**) for 1000 nm; (**d**–**f**) for 1500 nm.

To explore the selective detection of skin depth using multi-SDSs, we proposed circular photosensitive area detectors with a radius of 0.1 mm, placed on the skin surface at specific separations from the light source. Specifically, we selected wavelengths of 1000 nm and 1500 nm, where hemoglobin and water exhibit strong absorption [37]. The proportion of the mean optical path in each skin layer relative to the total mean optical path is shown in Figure 3. We plotted the β values for all skin layers.

To ensure that $\beta_{Dermis}$ exceeds 85% at each wavelength, the SDS must be at least 1.7 mm. At 1000 nm, the SDS greater than 2.9 mm results in the involvement of $\beta_{Subcutaneous}$. Therefore, to focus the detection range on the dermis while minimizing subcutaneous tissue interference, the SDS between 1.7 mm and 2.9 mm is appropriate. In vivo skin measurements can be influenced by fluctuations in cutaneous blood perfusion, which may interfere with the upper and deep blood net dermis [38]. Monitoring or avoiding these impacts is crucial. As shown in Figure 3b and Figure 3e, the SDS of 1.7 mm is suitable for monitoring changes in the upper blood net dermis, with $\beta_{Upper\_blood}$ values of 10% and 29% at 1000 nm and 1500 nm, respectively. Additionally, using a smaller SDS (e.g., SDS = 1.7 mm) minimizes the influence of the deep blood net dermis, with $\beta_{Deep\_blood}$ values below 0.2%.

**Figure 4:**
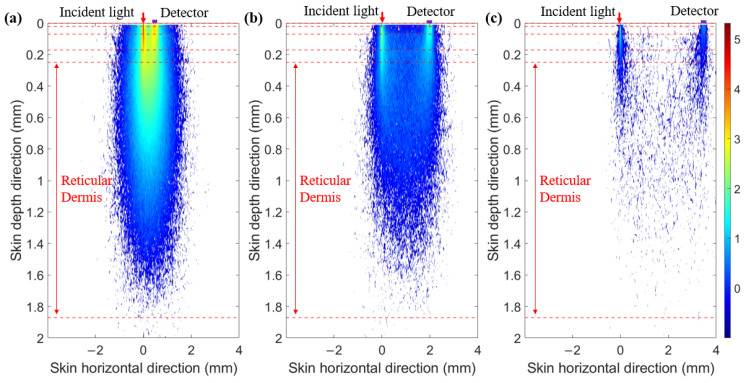
Optical field at 1000 nm. (**a**) SDS of 0.5 mm. (**b**) SDS of 2.0 mm. (**c**) SDS of 3.5 mm.

Next, we plotted the optical field in multi-SDSs at 1000 nm (Figure 4). We found that at a smaller SDS, light energy is more concentrated in the superficial layers of the skin. As the SDS increases, the light energy penetrates deeper into the skin layers, although its intensity decreases. Therefore, SDS can effectively select information from different depth ranges within the skin.

### 3.2. Design Proposal for Detection SNR

#### 3.2.1. Detection SNR Enhancement by Increasing the Photosensitive Area

**Figure 5:**
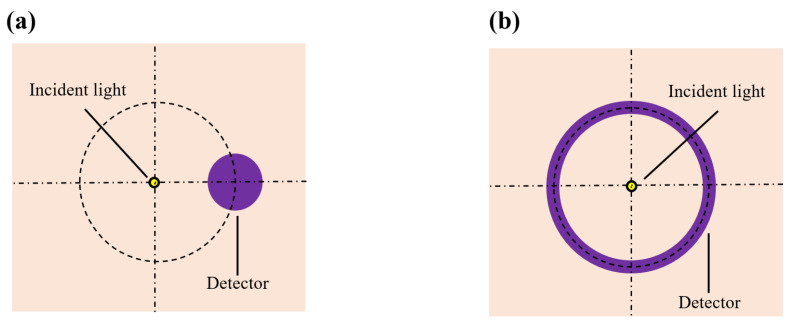
Top view of sensors with various photosensitive area shapes: (**a**) is the circular photosensitive area; (**b**) is the ring-shaped photosensitive area.

**Figure 6:**
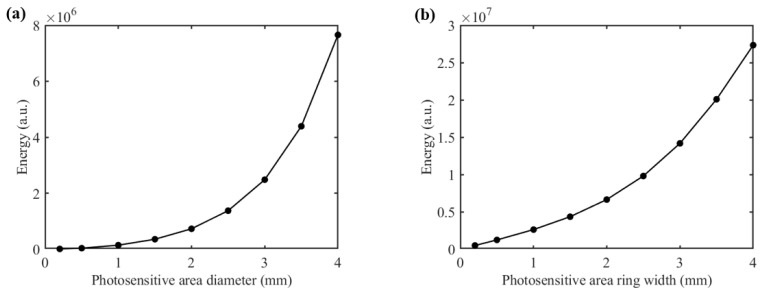
Light energy distribution of diffuse reflection light at 1000 nm: (**a**) for the circular photosensitive area; (**b**) for the ring-shaped photosensitive area.

The shape and size of the detector’s photosensitive area play a critical role in determining both the detection SNR and depth resolution. We proposed two different shapes for the detector’s photosensitive area: a circular area and a ring-shaped area (Figure 5a and Figure 5b, respectively). With the SDS fixed at 2.3 mm and at 1000 nm, we varied the sizes of both types of photosensitive areas and recorded the corresponding magnitude of the detected light energy, as shown in Figure 6. It is evident that as the size of the photosensitive area increases, the detected light energy also increases. For instance, when the diameter of the circular photosensitive area was increased from 1 mm to 2 mm, the detected light energy increased by a factor of 5.19. Similarly, when the width of the ring-shaped area was increased from 1 mm to 2 mm, the detected light energy increased by a factor of 2.54. Moreover, the ring-shaped photosensitive area can receive more light energy. Notably, while the size of the photosensitive area increased, the associated rise in noise level was much smaller than the increase in detected signal level, indicating that enlarging the photosensitive area can significantly improve the detection SNR.

#### 3.2.2. Shape Design of the Detector

**Figure 7:**
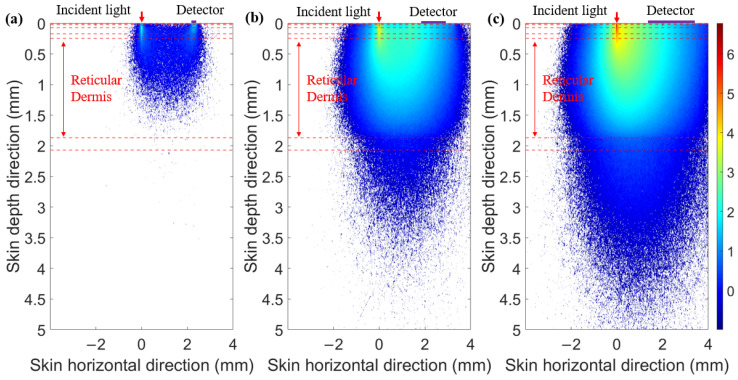
Optical field for circular photosensitive areas of different diameters at 1000 nm: (**a**) diameter of 0.1 mm, (**b**) diameter of 1 mm, and (**c**) diameter of 2 mm.

**Figure 8:**
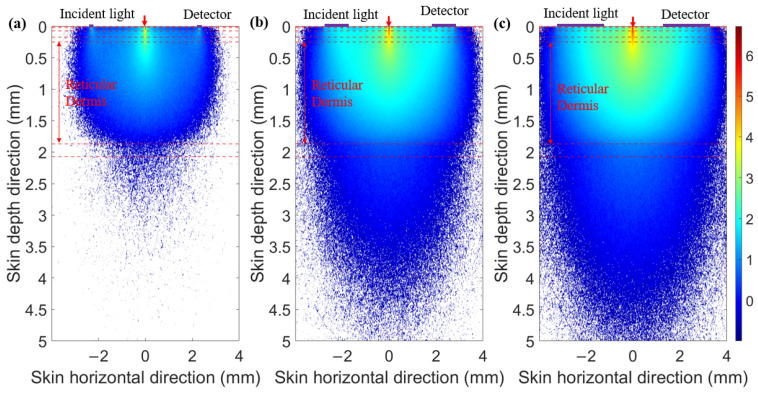
Optical field for ring-shaped photosensitive areas of different ring widths at 1000 nm: (**a**) ring width of 0.1 mm, (**b**) ring width of 1 mm, and (**c**) ring width of 2 mm.

When the SDS is set to 2.3 mm, we compared the optical field distribution of two detector shapes (circular and ring-shaped), as shown in Figure 7 and Figure 8, respectively. The results demonstrate that the optical field of the ring-shaped photosensitive area is more uniform than that of the circular one. The ring-shaped area collects more light energy and has the added advantage of gathering light from multiple directions on the skin. This results in a more uniform optical field across different receiving directions, providing a more consistent depth resolution for the same SDS.

**Figure 9:**
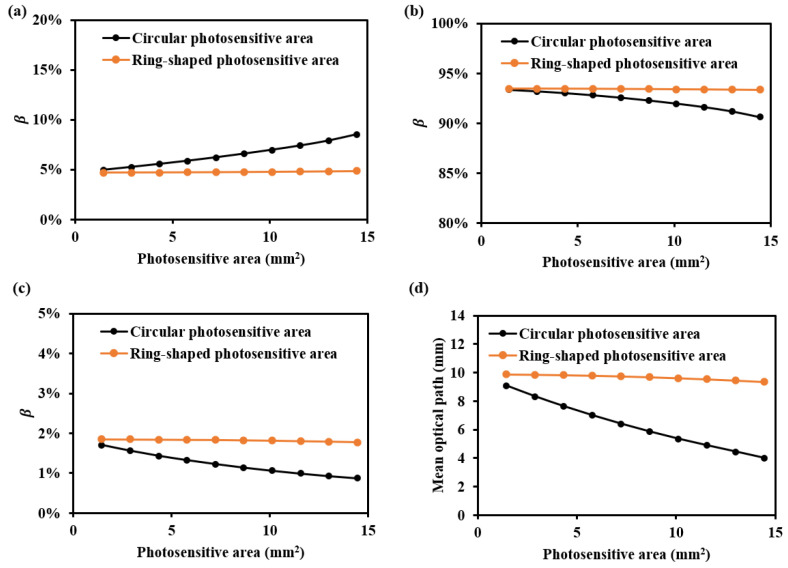
β and mean optical path for various shapes of photosensitive areas at 1000 nm: (**a**) $\beta_{Epidermis}$, (**b**) $\beta_{Dermis}$, (**c**) $\beta_{Subcutaneous}$, and (**d**) mean optical path.

The β values for various shapes of photosensitive areas at 1000 nm are shown in Figure 9. It can be observed that as the detector’s photosensitive area increases, the $\beta_{Dermis}$ is higher for the ring-shaped photosensitive area. Additionally, the mean optical path decreases more slowly with an increase in the photosensitive area of the ring-shaped area. This is because, with a larger photosensitive area, the circular photosensitive area tends to receive more information from the nearby light source, thereby reducing the received optical path and depth resolution. In contrast, the ring-shaped photosensitive area, with its higher and more evenly distributed depth resolution across multiple directions, offers a better design guide for enhanced performance.

#### 3.2.3. Design of the Ring-Shaped Detector

For a ring-shaped detector, its SDS and corresponding ring width are designable factors. Generally, for a given SDS, the narrower the ring width, the more precise the corresponding detection depth, resulting in a better depth resolution. However, a narrower ring width also leads to weaker light intensity. Therefore, the design principle is to minimize the ring width as much as possible while ensuring sufficient light intensity.

(1) Design of ring-shaped detectors for the dermis

**Figure 10:**
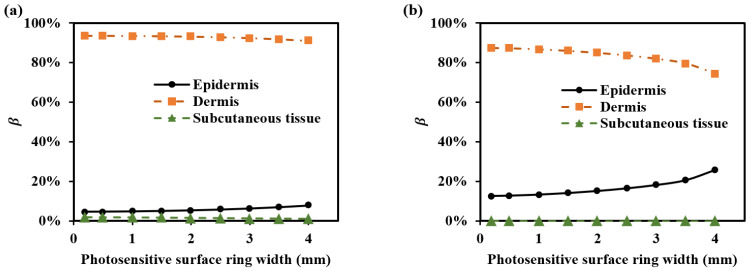
β for various ring widths of the ring-shaped photosensitive area: (**a**) 1000 nm and (**b**) 1500 nm.

Based on the simulation results in Section 3.1.2, the results indicate that more than 85% of the optical path at the SDS in the range of 1.7–2.9 mm passes through the dermis. Thus, the detection depth within this SDS range primarily is in the dermis. Therefore, when selecting SDS and its corresponding ring width from the perspective of the optical path, there are few restrictions. For instance, in Figure 10, at a SDS of 2.3 mm, simulations of multiple ring widths ranging from 0.2 mm to 1.4 mm reveal that over 85% of the optical path originates from the dermis. Therefore, variations in the ring width have little impact on detecting the dermis in this context. The minimum recommended value for the ring width is 0.2 mm, as we developed a good-performance sensor with a 0.2 mm ring width for five SDSs ranging from 1.7 to 2.9 mm [8,10].

(2) Design of ring-shaped detectors for the multiple sub-layers within the dermis

Due to the relatively thick and complex structure of the dermis, as well as the non-uniform diffusion of BGC in skin, obtaining information from different depths within the dermis requires the improved SDS’s depth resolution. As shown in Figure 1, the dermis is divided into four sub-layers (papillary layer, upper blood net dermis, reticular dermis, and deep blood net dermis). The reticular dermis, being relatively thick, can be further subdivided. We recommend a minimum ring width of 0.2 mm. To more precisely observe changes in the four sub-layers, the SDS range of 1.7–2.9 mm is divided into different independent parts, as shown in Table 2. It includes five-ring detectors, four-ring detectors, and three-ring detectors. The results from these different detectors can be combined to increase the light intensity or used separately when a sufficient light intensity is available.

**Figure 11:**
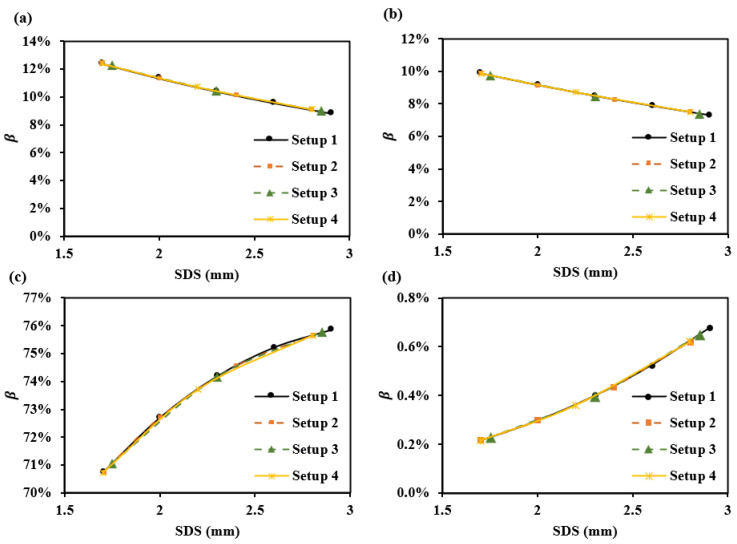
β for various setups at 1000 nm: (**a**) $\beta_{Papillary\;dermis}$, (**b**) $\beta_{Upper\;blood\;net\;dermis}$, (**c**) $\beta_{Reticular\;dermis}$, and (**d**) $\beta_{Deep\;blood\;net\;dermis}$.

Figure 11 shows the optical path proportions for each SDS corresponding to the four sub-layers of the dermis in these setups (Table 2). It can be observed that smaller SDSs, such as 1.7 mm, have a higher $\beta_{Papillary\;dermis}$ and $\beta_{Upper\;blood\;net\;dermis}$, while larger SDSs, such as 2.9 mm, have a higher $\beta_{Reticular\;dermis}$ and $\beta_{Deep\;blood\;net\;dermis}$.

**Figure 12:**
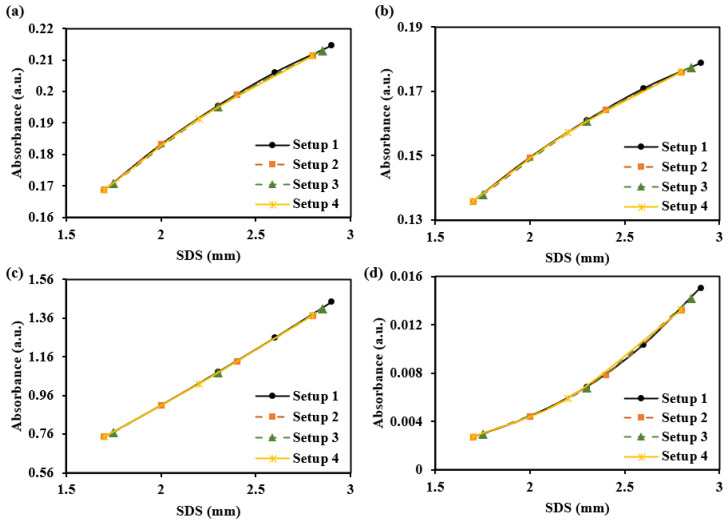
The absorbance changes caused by a 2 cm^−1^ increase in the absorption coefficient of each dermal layer at 1000 nm: (**a**) for the papillary dermis, (**b**) for the upper blood net dermis, (**c**) for the reticular dermis, and (**d**) for the deep blood net dermis.

Figure 12 simulates the sensing capabilities of these different SDSs for dermal information at different depths. We assume the absorption coefficient increases by 2 cm^−1^ in the papillary dermis, upper blood net dermis, reticular dermis, and deep blood net dermis. The results show that, with increasing SDS, the response slopes of absorbance changes vary across different layers. Specifically, changes in the superficial dermis exhibit steep-to-gentle absorbance variations, while changes in the deeper dermis show gentle-to-steep absorbance variations. The changes in the central region of the dermis are relatively uniform. Based on the simulation, it was observed that positioning 3–5 SDSs within a range of 1.7–2.9 mm enables the detection of changes across various layers. The more SDSs are set, the more details can be captured along the absorbance curve corresponding to the SDS. Table 3 provides recommendations for both a single SDS and a set of SDSs configurations.

### 3.3. Design Proposal for Human–Sensor Interface Coupling

#### 3.3.1. Non-Contact Skin and Detector

**Figure 13:**
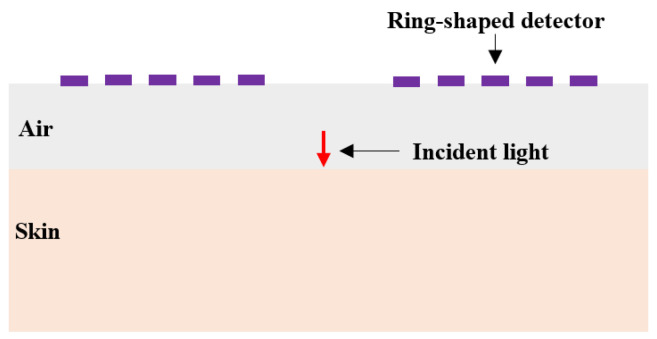
Schematic diagram of SDD design.

**Figure 14:**
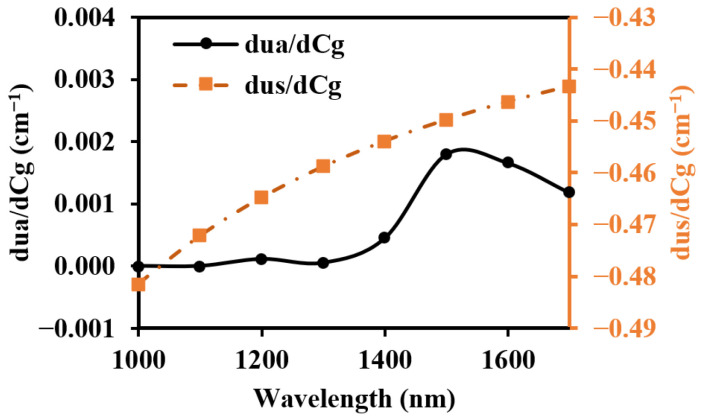
Optical parameter changes induced by the 1 mM BGC.

**Figure 15:**
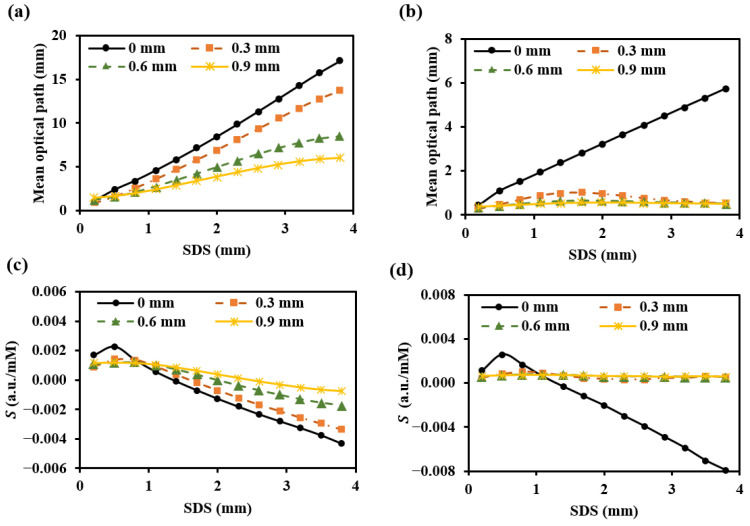
Mean optical path (**a**,**b**) and BGC sensitivity (**c**,**d**) for different SDDs: (**a**,**c**) for 1000 nm; (**b**,**d**) for 1500 nm.

**Figure 16:**
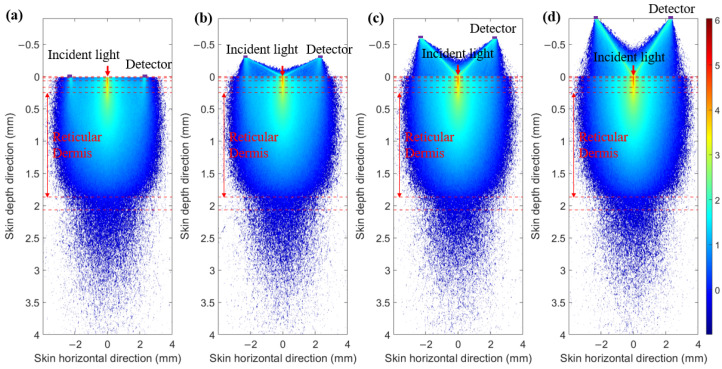
Optical field at 1000 nm. (**a**) SDD of 0 mm. (**b**) SDD of 0.3 mm. (**c**) SDD of 0.6 mm. (**d**) SDD of 0.9 mm.

To prevent contamination caused by direct contact between the skin and detectors, and to ensure a certain level of breathability at the interface, it is crucial to design an appropriate skin–detector distance (SDD) for practical applications (Figure 13). However, this design often leads to spatial crosstalk between detectors, which can affect the optical path detected by the detectors. As shown in Figure 14, the changes in the optical parameters induced by BGC variations were based on the results from Larin et al. [39] and Ge et al. [10]. The change in the scattering coefficient caused by the BGC is dominant, leading to a negative variation in absorbance [8,10]. We considered four SDDs: 0, 0.3, 0.6, and 0.9 mm. Using MC simulations, we explored the impact of these distances on the optical path and BGC sensitivity (Figure 15). As the SDD increases, the mean optical path gradually decreases, resulting in a reduction in BGC sensitivity. This distance directly influences the response of the detection signal. We also plotted the optical field at different SDDs at 1000 nm (Figure 16). It was observed that emergent light near the light source is the primary interference, which causes spatial crosstalk between the detectors.

#### 3.3.2. Design to Prevent Crosstalk of Spatial Light Between Detectors

**Figure 17:**
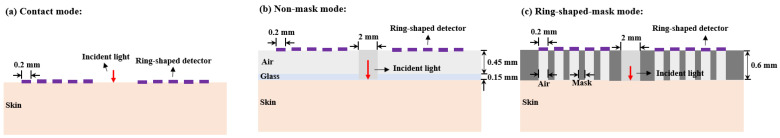
Three skin–sensor interface modes. (**a**) Contact mode. (**b**) Non-mask mode (Han et al. [8]). (**c**) Ring-shaped-mask mode.

When considering an appropriate SDD, it is essential to utilize spatial mask structures to minimize interference from light emitted near the source. This approach aims to achieve a detection performance comparable to that of direct contact. To prevent crosstalk from the incident light, we proposed a guiding sleeve piece for light transmission [8]. The sleeve piece has a radius of 1 mm. We compared three skin–sensor interface modes (Figure 17). The contact mode (Figure 17a) represents the detection effect at zero SDD. The non-mask mode (Figure 17b), which includes spatial crosstalk, features an interface composed of a 0.45 mm air layer and a 0.15 mm glass layer. In the design by Han et al. [8] and Ge et al. [10], the non-mask mode was adopted. In this mode, the sensor demonstrated preferable glucose signal detection capabilities during oral glucose tolerance tests (OGTTs). The ring-shaped-mask mode (Figure 17c) exposes only the measurement area directly beneath the photosensitive area. The absorption coefficient of the mask and sleeve material was set to infinity, ensuring that all photon energy was attenuated when transmitted to the edge of the mask.

**Figure 18:**
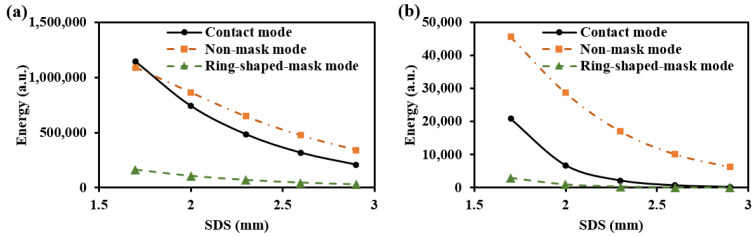
The light energy distribution across three skin–sensor interface modes: (**a**) for 1000 nm and (**b**) for 1500 nm.

**Figure 19:**
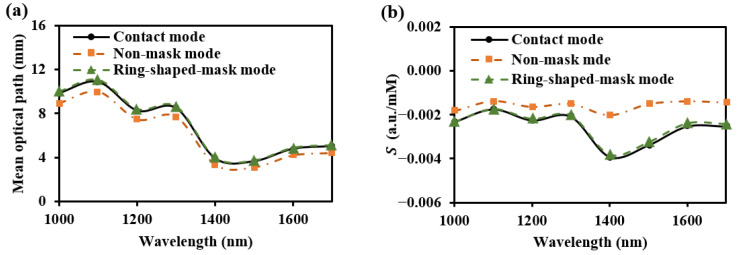
Mean optical path (**a**) and BGC sensitivity (**b**) at the three skin–sensor interfaces. (SDS = 2.3 mm).

**Figure 20:**
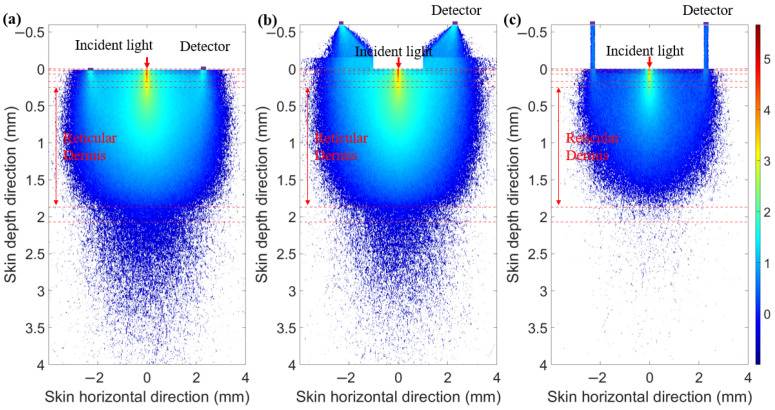
Optical field for three skin–sensor interface modes. (**a**) Contact model. (**b**) Non-mask mode (Han et al. [8]). (**c**) Ring-shaped-mask mode.

As shown in Figure 18, the light energy distribution across three skin–sensor interface modes reveals that the ring-shaped-mask mode reduces light energy by approximately 85% compared to the contact mode. This structure results in some loss of the SNR, but enhances the depth resolution. Taking SDS = 2.3 mm as an example, the mean optical path and BGC sensitivity for the three skin–sensor interface modes are shown in Figure 19. The results indicate that the mean optical path and BGC sensitivity in the ring-shaped-mask mode are similar to those in the contact mode. In contrast, the non-mask mode exhibits a shorter optical path and reduced BGC sensitivity. Therefore, the ring-shaped-mask mode effectively prevents the interference from spatial crosstalk light between detectors. It also enhances the depth resolution. The optical field for the three skin–sensor interface modes are shown in Figure 20. It can be observed that the non-mask mode receives more emergent light from the nearby light source, while the ring-shaped-mask mode detects photons primarily from the skin surface directly beneath the detector.

### 3.4. Available Design Proposal for Human Detection

#### 3.4.1. Differential Measurement Strategy on Humans

Based on our human experiment experience, the single SDS detector exhibits high $\sigma I_{human}$, resulting in a low SNR that fails to meet the measurement requirements. In contrast, adopting the differential measurement strategy by using two SDSs significantly reduces $\sigma I_{human}$. Experiments by Han et al. [8] and Ge et al. [10] have demonstrated that the differential measurement strategy achieves a preferable performance in OGTTs, successfully detecting optical signals that vary with the BGC at 1550 nm. Therefore, we recommend selecting two appropriate SDSs for human measurements. Similar to the design principles for the single SDS, the SNR and BGC sensitivity of the sensor, after differential processing, must also meet the requirements for detecting the desired $C_{g,limit}$ of the BGC.

The differential absorbance under two SDSs is defined as:

(11) $$A_{D}\left(\lambda \right)=A\left(\lambda ,\rho_{B}\right)-A\left(\lambda ,\rho_{A}\right)$$

where the two SDSs are $\rho_{A}$ and $\rho_{B}$ with $\rho_{B}>\rho_{A}$. Many interferences cannot be fully mitigated by the sensor alone. Therefore, it is necessary to employ mathematical methods to eliminate unwanted interferences [40,41]. At the incident light source, the differential measurement strategy eliminates interference caused by drift in the incident light source [8].

Correspondingly, we define the equivalent differential SNR as:

(12) $${SNR}_{D}=\left|\frac{1}{\sigma A_{D}}\right|$$

The sensitivity of the differential absorbance to the BGC is denoted as $S_{D}$.

(13) $$S_{D}=\frac{dA_{D}\left(\lambda ,\rho \right)}{dC_{g}}$$

The $C_{g,limit}$ can be expressed as:

(14) $$C_{g,limit}=\frac{1}{\left|S_{D}\cdot {SNR}_{D}\right|}=\left|\frac{\sigma A_{D}}{\frac{dA_{D}\left(\lambda ,\rho \right)}{dC_{g}}}\right|$$

Once the BGC sensitivity is determined, the SNR_D_ should meet the requirement, as follows:

(15) $${SNR}_{D}\geq \frac{1}{\left|S_{D}\cdot C_{g,limit}\right|}$$

In the experiments by Han et al. [8] and Ge et al. [10], when the SDSs were 1.7 and 2.0 (with a ring width of 0.2 mm), the sensor’s SNR_D_ was approximately 1429:1–5000:1 within 1 h. Based on the value $S_{D}$ around 0.001 a.u., it can be deduced that the $C_{g,limit}$ reached 0.2–0.7 mM.

#### 3.4.2. Available SDSs for Differential Measurements

For the differential measurement strategy, we optimized the two selected SDSs and their ring widths. The design principles for the two ring-shaped detectors are as follows:

(1) The optical paths corresponding to the two SDSs should predominantly pass through the dermis.
(2) The optical path difference between the two SDSs should be as large as possible.
(3) The SNR_D_ and $S_{D}$ of the two SDSs should meet the requirements of Equation (14) to acquire the desired $C_{g,limit}$.

Therefore, this paper provides optional proposals for the differential measurement of the dermis, as shown in Table 4.

**Figure 21:**
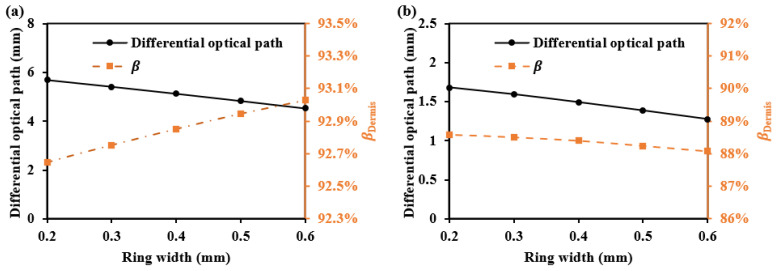
The differential optical path and $\beta_{Dermis}$ at the second SDS when using two-ring detectors: (**a**) for 1000 nm and (**b**) for 1500 nm.

For whole dermis detection, we offer two detectors with the biggest SDS difference for the two SDSs. These configurations effectively enhance the differential optical path, making it suitable for detecting signals over the whole dermis. When the first SDS is fixed at 1.7 mm with a ring width of 0.2 mm, the second SDS and ring width are varied. Figure 21 shows the second SDS’s $\beta_{Dermis}$ for each ring width, as well as the average optical path difference between the two SDSs. The results show that as the ring width increases, the differential optical path becomes smaller, and the $\beta_{Dermis}$ values for different ring widths are all above 88%.

**Figure 22:**
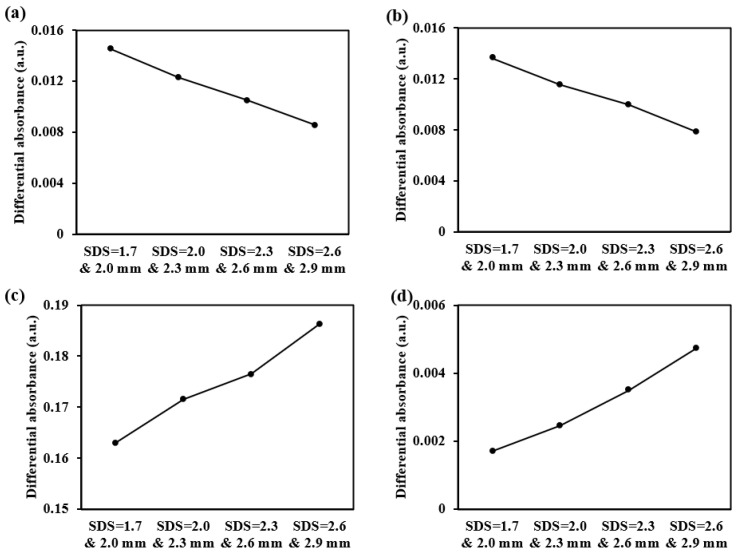
The differential absorbance changes caused by a 2 cm^−1^ increase in the absorption coefficient of each dermal layer at 1000 nm: (**a**) for the papillary dermis, (**b**) for the upper blood net dermis, (**c**) for the reticular dermis, and (**d**) for the deep blood net dermis.

For the sub-layers of the dermis detection, we offer more detectors and employ a differential strategy between adjacent detectors. This approach enhances the depth resolution of detection. Taking five-ring detectors as an example, when the absorption coefficient of the four sub-layers of the dermis changes by 2 cm^−1^, the resulting differential absorbance changes are shown in Figure 22. The results demonstrate significant variations in the interference from different sub-layers in the differential absorbance. Specifically, in the superficial dermis, smaller SDS differences result in greater changes in the differential absorbance, whereas in the deeper dermis, larger SDS differences result in greater changes in the differential absorbance.

## 4. Performance Testing for Two Sensors with Five-Ring Detectors

### 4.1. Measurement System and Experiment Arrangements

We made two sensors (the non-mask mode sensor in Figure 17b and ring-shaped-mask mode sensor in Figure 17c) composed of five-ring detectors and tested their noise levels, including the SNR and SNR_D_ for the SDSs of 2.0 mm and 2.6 mm. Based on the BGC sensitivity obtained from MC simulations, we estimated the potential detection limits of the BGC achievable with the sensors. Both the sensors feature five separate ring-shaped detectors (each with a ring width of 0.2 mm) arranged concentrically around the incident fiber, with SDSs at 1.7, 2.0, 2.3, 2.6, and 2.9 mm. Among these, we selected two SDSs (2.0 and 2.6 mm) for differential measurements due to their preferable SNRs. The SDS of 2.9 mm was not used, as its light intensity was significantly reduced after applying the mask for the sensor in Figure 17c.

**Figure 23:**
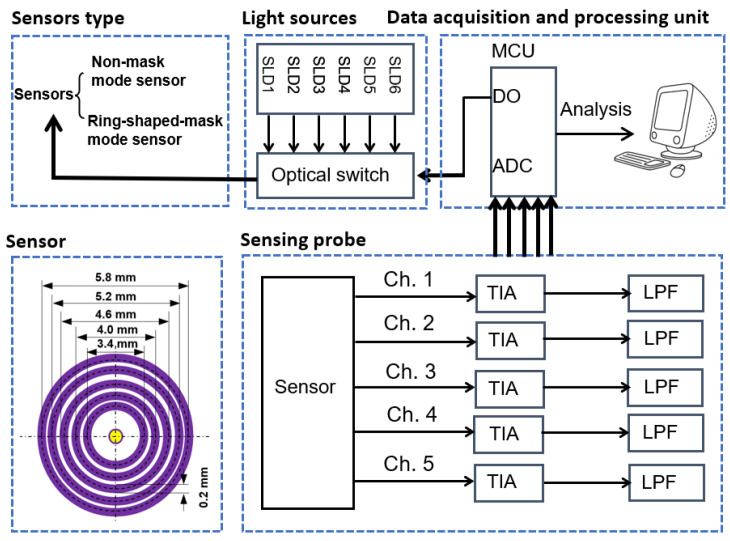
Experimental measurement system.

The measurement system is shown in Figure 23. The subject’s forearm (Asian male, 25 years old) and a 20% calibrated diffuse reflectance standard (RSS-08-010, Labsphere, North Sutton, NH, USA) were tested. The experimental setup included a multi-SDS InGaAs sensor, six superluminescent diodes (with center wavelengths of 1050 nm, 1219 nm, 1314 nm, 1380 nm, 1550 nm, and 1609 nm, and 3 dB bandwidths of 51 nm, 32 nm, 36 nm, 58 nm, 52 nm, and 57 nm), an optical switch, and a data acquisition and processing unit. Two sensors (a non-mask mode sensor and ring-shaped-mask mode sensor) were assembled into the measurement system. We evaluated the sensors at 1550 nm, as this wavelength offers greater BGC sensitivity [8,10]. The ambient temperature during the test was maintained in the range of 24–25 °C to ensure the stability of the detector’s thermal noise. Before testing, preheat the light source for at least 3 h to prevent its drift.

Firstly, we tested the sensor’s noise levels using a 20% calibrated diffuse reflectance standard. The diffuse reflectance standard was placed approximately 1 cm away from the sensor. Next, the two sensors were tested on human forearm skin, ensuring the subject was fasting for at least 5 h. Additionally, during the human measurement, the dark noise (caused by the dark current) was recorded at the beginning of each light intensity collection. All measurement data deducted the dark noise.

### 4.2. Test Results

**Figure 24:**
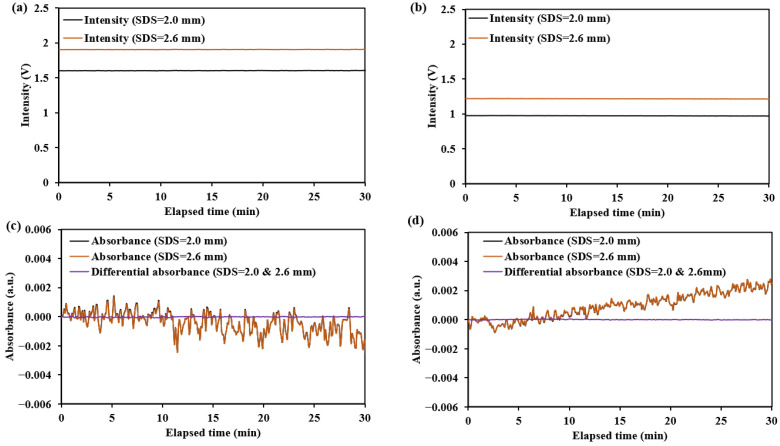
The light intensity and absorbance change results for the diffuse reflectance standard at 1550 nm: (**a**,**c**) for the non-mask mode sensor (Han et al. [8]); (**b**,**d**) for the ring-shaped-mask mode sensor.

**Figure 25:**
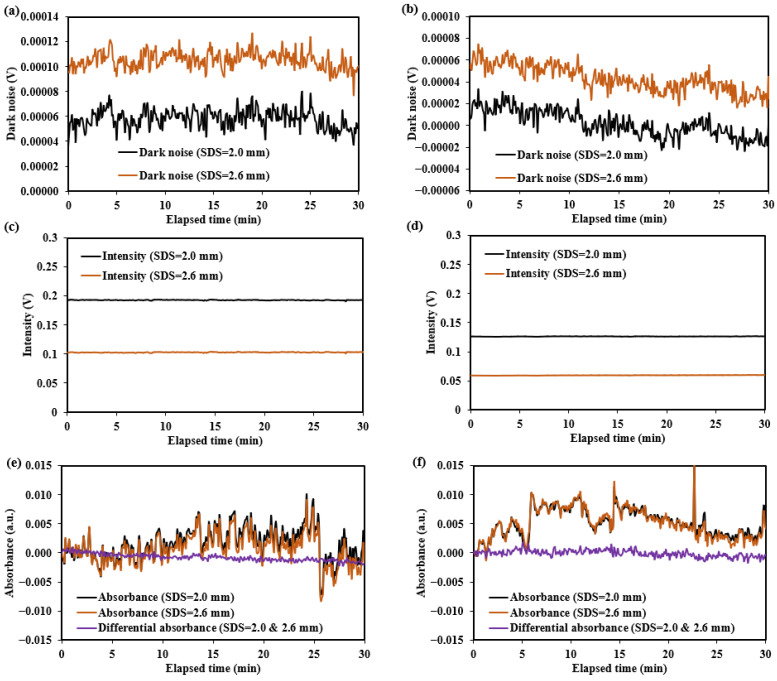
Dark noise, light intensity, and absorbance change results for human forearm skin at 1550 nm: (**a**,**c**,**e**) for the non-mask mode sensor (Han et al. [8]); (**b**,**d**,**f**) for the ring-shaped-mask mode sensor.

The test results for the diffuse reflectance standard and human forearm skin are shown in Figure 24 and Figure 25, with the summarized numerical data presented in Table 5.

The dark noise and diffuse reflectance light intensity on human forearm skin are recorded in Figure 25. The results show that dark noise is relatively low, ranging from −0.00004 V to 0.00013 V, accounting for approximately 0.008–0.1% of the detected light intensity (0.05–0.2 V). These dark noises are primarily composed of near-infrared radiation from the environment and the human skin. Therefore, additional calibration of the sensors is required to enable more accurate measurements on the human body.

The noise for the diffuse reflectance standard primarily originates from $\sigma I_{detector}$, which mainly reflects the thermal noise of the detector. The noise observed during human testing primarily reflects $\sigma I_{human}$, which is mainly related to factors such as the jitter of the measurement site and the non-uniform skin surface reflection. The fluctuations in absorbance or differential absorbance observed in Figure 24 and Figure 25 during the testing period reflect the magnitude of σA. As indicated by Equation (4), a smaller σA corresponds to a higher SNR.

The testing results for the two sensors (the non-mask mode sensor and ring-shaped-mask mode sensor) indicate the ring-shaped-mask mode sensor exhibits a significant reduction in light intensity and lower SNR and SNR_D_. Moreover, the SNR and SNR_D_ evaluated on the human skin are significantly lower than those on the diffuse reflectance standard for both the sensors.

In human skin testing, changes in the absorbance and differential absorbance at 1550 nm were recorded over the 30 min period, as shown in Figure 25. Significant drift and fluctuation in the absorbance changes were observed, while the use of differential absorbance improved the signal stability. For the non-mask mode sensor, the SNR was 377:1, and the SNR_D_ was 1732:1 over 30 min. For the ring-shaped-mask mode sensor, the SNR was 321:1, and the SNR_D_ was 1366:1 over 30 min. The differential measurement strategy enhances the SNR in human body measurements. The detection optical path may lose some values due to the differential processing, but the depth resolution is improved. After differential processing, the detection depth becomes more focused within a specific range. Overall, the differential measurement strategy improves detection effectiveness.

The ring-shaped-mask mode sensor reduces the detected light intensity, resulting in a lower SNR compared to the non-mask mode sensor. However, the MC simulations show that the BGC sensitivity of the ring-shaped-mask mode sensor is approximately twice that of the non-mask mode sensor. Based on the estimates of $C_{g,limit}$ from Equations (6) and (14), the ring-shaped-mask mode sensor demonstrates a preferable BGC detection performance, with $C_{g,limit}$ derived from the differential absorbance of approximately 0.37 mM.

## 5. Discussion and Conclusions

We proposed a comprehensive discussion on sensor design from three key perspectives: depth resolution, detection SNR, and human–sensor interface coupling. Additionally, we presented design proposals that demonstrated preferable results, but still offer opportunities for further optimization.

To achieve the necessary depth resolution, we proposed a multi-SDS design ranging from 1.7 to 2.9 mm, applicable to the wavelength range of 1000 to 1700 nm. This design meets the requirements for multi-layer detection depths at different wavelengths. Within this SDS range, the $\beta_{Dermis}$ value is relatively high (exceeding 85%), which helps enhance the detection capability for BGC signals. However, to accommodate broader and more precise detection needs, the SDS design requires further enhancement. For instance, extending the SDS range can address the detection depth requirements of different individuals, measurement areas, and wavelengths. A smaller SDS (<1.7 mm) can be added for detecting changes in the epidermal water content, while a larger SDS (>2.9 mm) is better suited for detecting changes in subcutaneous tissue. Similarly, optimizing SDS for wavelengths with strong water absorption (e.g., 1400 nm) can improve the detection of changes in interstitial fluid water content. Moreover, the ring width of a single SDS can also be optimized. Narrower ring widths correspond to more precise depth information. Due to the relatively thick dermis, the requirements for ring width design are less stringent, with ring widths ranging from 0.2 to 1.4 mm being suitable. However, the dermis is subdivided into the papillary dermis, upper blood net dermis, reticular dermis, and deep blood net dermis. By detecting at different depths of the dermis, multiple SDSs can be set within the range of 1.7–2.9 mm, with each SDS having a various ring width. Table 3 presents our recommended results. Based on our recommendations, three to five SDSs can be configured for detecting the dermal sub-layers. This proposed design can also detect the uneven diffusion of BGC from the blood vessels to interstitial fluid. The light signals across different SDSs provide a good basis for determining the depth of variables. Additionally, multi-ring detectors can be flexibly combined. When the light source intensity is weak, merging the signals of adjacent rings can enhance the light intensity and improve detection efficiency.

To achieve enough detection SNRs, the design proposed in this paper involves using a photodiode with a ring-shaped detection area placed close to the skin to directly receive light. This design ensures that photons have the same SDS in all directions, significantly increasing the detection area compared to traditional circular photosensitive area. It improves both the depth resolution and SNR. And the continuous ring-shaped photosensitive area averages the anisotropic interference from the human body as well as variations in the incident light spot density. Compared to light reception from a ring-shaped fiber bundle, the InGaAs ring-shaped detector, with its larger photosensitive area and higher receiving efficiency, demonstrates superior detection capability. However, the manufacturing complexity of the ring-shaped photosensitive area and its adaptability to uneven measurement surfaces still need further optimization. For example, exploring other shapes, such as circular or arc-shaped photosensitive areas, could better accommodate diverse measurement regions. Furthermore, to enable effective human measurements, we recommend using a differential measurement strategy, selecting two SDSs. We provided the principles and proposals for selecting two SDSs to measure the dermis (Table 4). Similarly, to enhance the detection of multiple sub-layers and varying depths within the dermis, multiple SDSs can be set (Table 4). The differential measurement strategy can be performed using two adjacent or close SDSs.

To achieve good human–sensor interface coupling, we proposed a design that maintains a fixed distance of approximately 0.6 mm between the skin and detectors, combined with a ring-shaped-mask structure (Figure 17c). This design reduces spatial crosstalk while achieving a performance comparable to direct skin contact. The experiments confirmed the preferable detection performance of this sensor, but further improvements in human–sensor interface coupling are still possible. The measurement area directly beneath the ring-shaped-mask structure is hollowed out to prevent sweat accumulation during short-term measurements. However, the area beneath the mask remains prone to sweat buildup during prolonged contact. Therefore, optimizing the mask material, such as using water-absorbing materials in direct contact with the skin, remains a key focus. While the ring-shaped-mask structure effectively reduces spatial crosstalk, it results in approximately 85% loss of detected light intensity. This loss can be compensated by combining multiple rings or increasing the incident light energy. Han et al. [8] and Ge et al. [10] tested the performance of sensors with non-mask mode structures (Figure 17b) in OGTTs, achieving satisfactory results. This study only conducted the preliminary performance testing of the sensor. Further OGTTs will be carried out using the ring-shaped-mask mode sensor.

Finally, we proposed a sensor for differential measurements. Five independent ring-shaped detectors were set within the SDS range of 1.7–2.9 mm, and a ring-shaped-mask structure was designed to effectively prevent the generation of spatial crosstalk light, achieving a preferable coupling interface between the skin and the sensor. Human testing results indicate that the SNR_D_ may meet the requirements for BGC detection.

To enable the sensor’s application in long-term daily use, several interferences arising from varying measurement conditions still need to be addressed. Firstly, the instrument requires regular calibration to eliminate measurement errors caused by instrument background drift and changes in human tissue background. These tissue background variations are complex and include factors such as fluctuations in the intrinsic near-infrared radiation from the human body, changes in body temperature, variations in tissue hydration, and dynamic cutaneous blood perfusion. Additionally, random displacements in measurement locations can affect data stability. To address this issue, a fixed positioning approach similar to that used for continuous glucose monitoring (CGM) could be employed, ensuring that the sensor maintains a consistent position over time, thereby enhancing the measurement stability. For non-uniform interference (such as uneven glucose diffusion and water distribution within the body), the multi-SDS ring-shaped sensor offers significant advantages. It provides rich depth-sensing information that helps to more accurately identify and differentiate various types of interference signals. At the same time, with the continuous advancement of artificial intelligence technology, the existing smart algorithms can analyze these multi-dimensional data. It can enable a more precise extraction of BGC signals and reduce interference effects.

In conclusion, to meet the technical requirements of NBGM, this study presents three design key points for diffuse reflectance optical sensors along with corresponding design proposals. Preliminary performance testing of the sensor was also conducted. This study is expected to provide references for the design of NBGM sensors.

## Figures and Tables

**Table 1:** Design key points of diffuse reflectance optical sensors.

Design Key Points	Design Requirements	Design Proposals
Depth resolution	To achieve the target detection depth, such as the detection depth mainly concentrated in the dermis.	Different SDSs are proposed to detect different target tissue depths across multi-wavelengths.
Detection SNR	To achieve the BGC signal resolution requirements, such as distinguishing a 1 mM BGC change.	Design of the detector’s photosensitive area shape and size.
	The measurement process allows the tissue to change within a certain range, such as being insensitive to a certain tissue deformation.	Differential measurement strategy.
Human–sensor interface coupling	Good detection and not easy to sweat, such as the sensor not being in contact with the skin and presenting the contact detection effect.	The skin–detector interface is air. Mask structure design to prevent spatial crosstalk between detectors.

**Table 2:** Setups of multi-type ring-shaped detectors.

	Type	SDS (mm)	Ring Width (mm)
Setup 1	Five-ring detectors	1.7, 2.0, 2.3, 2.6, 2.9	0.2, 0.2, 0.2, 0.2, 0.2
Setup 2	Four-ring detectors	1.7, 2.0, 2.4, 2.8	0.2, 0.2, 0.3, 0.4
Setup 3	Three-ring detectors	1.75, 2.3, 2.85	0.3, 0.3, 0.3
Setup 4		1.7, 2.2, 2.8	0.2, 0.3, 0.4

**Table 3:** Recommendations for configuring a single SDS and a set of SDSs.

	Single SDS	A Set of SDSs
SDS (mm)	1.7–2.9	3–5 SDSs within the range of 1.7–2.9 (In Table 2)
Ring width (mm)	0.2–1.4	0.2–0.4

**Table 4:** Optional proposals for differential measurement conditions of the dermis.

Application Scenario	Type	ID	SDS (mm)	Ring Width (mm)	Optional Two SDSs for Differential Measurement
Whole dermis detection	Two-ring detectors	SDS#1	1.7	0.2	SDS#1&SDS#2
		SDS#2	2.7–2.9	0.6 (for SDS = 2.7) 0.2 (for SDS = 2.9)	
Sub-layers for dermis detection	Three-ring detectors	SDS#1	1.7	0.2	SDS#1&SDS#2 SDS#2&SDS#3
		SDS#2	2.2	0.3	
		SDS#3	2.8	0.4	
	Four-ring detectors	SDS#1	1.7	0.2	SDS#1&SDS#2 SDS#2&SDS#3 SDS#3&SDS#4
		SDS#2	2.0	0.2	
		SDS#3	2.4	0.3	
		SDS#4	2.8	0.4	
	Five-ring detectors	SDS#1	1.7	0.2	SDS#1&SDS#2 SDS#2&SDS#3 SDS#3&SDS#4 SDS#4&SDS#5
		SDS#2	2.0	0.2	
		SDS#3	2.3	0.2	
		SDS#4	2.6	0.2	
		SDS#5	2.9	0.2	

**Table 5:** Sensors’ test results at 1550 nm.

	Non-Mask Mode Sensor		Ring-Shaped-Mask Mode Sensor	
	Single SDS (2.0 mm)	Differential SDSs (2.0&2.6 mm)	Single SDS (2.0 mm)	Differential SDSs (2.0&2.6 mm)
The 30 min SNR for the diffuse reflectance standard	1341:1	46,783:1	1101:1	55,751:1
The 30 min SNR for human forearm skin	377:1	1732:1	321:1	1366:1
Simulated BGC sensitivity (a.u./mM)	−0.0012	−0.001	−0.0023	−0.002
Estimated limit detection accuracy (mM)	2.21	0.58	1.35	0.37

## Data Availability

The original contributions presented in the study are included in the article; further inquiries can be directed to the corresponding authors.
